# Acquisition and Elimination of Bacterial Vaginosis During Pregnancy: A Danish Population-Based Study

**DOI:** 10.1155/IDOG/2006/94646

**Published:** 2006-05-25

**Authors:** Ida Vogel, Poul Thorsen, Bernard Jeune, Bo Jacobsson, Niels Ebbesen, Magnus Arpi, Annie Bremmelgaard, Birger R. Møller

**Affiliations:** ^1^NANEA, Department of Epidemiology, Institute for Public Health, University of Aarhus, 8000 Aarhus C, Denmark; ^2^Department of Clinical Genetics, Aarhus University hospital, 8000 Aarhus C, Denmark; ^3^Institute of Public Health, University of Southern Denmark, 5000 Odense C, Denmark; ^4^Perinatal Center, Department of Obstetrics and Gynecology, Sahlgrenska Academy, Göteborg University, 405 30 Göteborg, Sweden; ^5^Department of Obstetrics and Gynecology, Odense University Hospital, 5000 Odense C, Denmark; ^6^Department of Clinical Microbiology, Frederiksberg Hospital, 2000 Frederiksberg, Copenhagen, Denmark

## Abstract

*Objectives*: the aim was to examine factors associated with acquisition and elimination of bacterial vaginosis in pregnancy. *Methods*: a group of 229 pregnant women were randomly selected from a population-based prospective cohort study of 2927. They were examined at enrollment (mean gestational weeks 16*w* + 0*d*) and again in mid-third trimester (mean gestational age 32*w* + 3*d*). *Measures*: BV (Amsel's clinical criteria), microbiological cultures of the genital tract and questionnaire data. *Results*: BV prevalence decreased from 17% in early second trimester to 14% in mid-third trimester due to a tenfold higher elimination rate (39%) than incidence rate (4%). Heavy smokers (> 10/*d*) in early pregnancy were at increased risk (5.3 [1.1–25]) for the acquisition of BV during pregnancy, as were women receiving public benefits (4.8 [1.0–22]), having a vaginal pH above 4.5 (6.3 [1.4–29]) or vaginal anaerobe bacteria (18 [2.7–122]) at enrollment. A previous use of combined oral contraceptives was preventive for the acquisition of BV (0.2 [0.03–0.96]). Elimination of BV in pregnancy tended to be associated with a heavy growth of *Lactobacillus* (3.2 [0.8–13]) at enrollment. *Conclusions*: acquisition of BV during pregnancy is rare and is associated with smoking, while the presence of anaerobe bacteria and a vaginal pH > 4.5 are interpreted as steps on a gradual change towards BV. In the same way heavy growth of *Lactobacillus* spp in early pregnancy may be an indicator of women on the way to eliminate BV.

## INTRODUCTION

Several studies have documented an increased risk for adverse pregnancy outcomes among women with infectious conditions of low pathogenicity like bacterial vaginosis [1, 2]. It is hypothesized that low virulence microbes may ascend into the intrauterine environment, initiating an inflammatory cascade, which in turn may precipitate preterm birth but also affecting the fetal growth. A recent animal study found mice pups exposed to intrauterine lipopolysaccharide at day 15 of a 19-20-day-long pregnancy to have a decreased body weight 14 days post partum. This suggests that intrauterine infections, besides a direct effect on the brain, may affect fetal as well as infant growth [3].

Bacterial vaginosis (BV) is characterized by a reduction in H_2_O_2_ producing lactobacilli and the resultant overgrowth of less favorable microbes creating a more pathogenic internal environment. A microbial foundation for bacterial vaginosis has been demonstrated in which *G vaginalis*, anaerobic bacteria, and *M hominis* constitute the pathologic core of bacterial vaginosis [4]. However, increased knowledge of prevalence and incidence rates of BV in pregnancy as well as of the risk factors associated with acquisition and elimination of BV in pregnancy is needed for a better understanding of the disease and its outcomes.

A characteristic feature of BV is that it changes over time, though only four observational studies on pregnant women examine the time course of BV during pregnancy [5–8]. 3 out of these 4 studies [5–7] show a decreasing prevalence as the pregnancy progresses using either Spiegel, clue cells, or Nugent as their definition for BV. In contrast herewith, one study [8] found an increase in prevalence of BV (Nugent). These findings indicate BV as a dynamic condition during pregnancy, and further that BV seems unlikely to be present in late pregnancy if absent in early pregnancy. The decrease in prevalence has been suggested to be associated with either increasing prevalence and amount of *Lactobacillus* spp [2] or decreasing sexual activity [5] as pregnancy progresses. Smoking, which is associated with the acquisition of BV in the non-pregnant [9], has not been addressed in pregnancy.

There are no longitudinal studies of BV in pregnancy using Amsel's clinical criteria as the definition of BV. The aims of the present study were to study the prevalence of bacterial vaginosis using the clinical definition at two times in pregnancy and to examine factors associated with acquisition and elimination of BV among a random selection of low-risk women drawn from a population-based study.

## MATERIALS AND METHODS

### Study population

A population-based prospective cohort study was performed with the purpose of studying bacterial vaginosis in pregnancy. The participants were pregnant women residing in the geographically defined catchment area (population about 240,000) of the Odense University Hospital, Denmark, who received routine prenatal care at the hospital from November 1992 to February 1994. To be accepted for the study, women had to have a prenatal visit before their 24th week of gestation (in Denmark, more than 97% of the pregnant population has received prenatal care by that point), be ≥ 18 years of age, able to understand Danish, planning to deliver at the study hospital, and not planning to move out of the country. In addition, for ethical reasons, we did not enrol any women with a history of severe fetal congenital malformation. Exclusion criteria were incomplete fulfillment of questionnaires, placenta previa (verified after 30 full gestational weeks), fetal loss, and delivery outside the present hospital. Of the 3596 eligible pregnant women, 3174 (88.4%) agreed to participate in the study and 2927 (81.4%) completed the study.

Details on data collection methods are described elsewhere [4, 10]. From the study base (*n* = 2927) 270 randomly selected participants were asked of a new pelvic examination in mid-third trimester, when they came for a routine checkup at the midwifery clinic. Of the 270 women 231 consented of an additional examination and sampling as at enrollment. Two of these women had received antibiotics efficient for eradicating BV and were excluded from all subsequent analyses, and the final study population thus consists of 229 women. The study was approved by the Regional Scientific Ethics Committee of Funen and Vejle County and the Danish Data Protection Agency in accordance with the Helsinki Declaration.

### Questionnaires

All participants were asked to fill in three questionnaires: (I) at enrollment, (II) at 30 weeks gestation, and (III) at birth. Questionnaire (I) consisted primarily of questions on previous and present reproductive and medical conditions. Questionnaire (II) dealt mostly with sociodemographic information and questionnaire (III) with urogenital and obstetric conditions. Finally, a registration form was filled in by the midwife for all participants shortly after birth. The following variable definitions were made on the basis of the questionnaires: low socio-economic status was defined as representing the two lowest socio-economic status groups V and VI (range I–VI). Single was defined as not having a husband/cohabitor. No higher education was defined as not having pursued formal education past the ninth grade. Infertility was defined as exceeding 24 months to conceive. A physically demanding job was defined as a job belonging to the highest of 4 categories on the basis of physical workload. Stressful life events were defined as two or more life events; family conflicts, violent episode(s) against the pregnant woman, worries about the unborn child, and concerns about the delivery. Serious medical disease was defined as insulin-dependent diabetes mellitus (IDDM), heart diseases, endocrine diseases, kidney diseases, or chronic infectious intestinal disease. High risk behavior was defined as driving without the use of a seat belt on a regular basis; this was interpreted as a general high risk-taking behavior. Sexual activity was measured as coitus frequency for the four weeks prior to fulfillment of the questionnaire. Heavy smoking was defined as smoking > 10 cigarets at enrollment and still smoking. Alcohol comsumption was dichotomized into 4 or more drinks per week at enrollment. Any previous use of combined oral contraceptives (yes, no). Any previous use of an intrauterine device (yes, no), or previous preterm birth (yes, no).

### Gestational age

Gestational age was confirmed by ultrasonographic measurements of the biparietal diameter and the femur length of the fetus at the 18th week of gestation among 97.5% of the participants. Gestational age was based upon last menstrual period for the remaining 2.5%.

### Microbiology

Vaginal samples for culture and BV assessment from all participants were collected from the posterior fornix after the vault of the vagina had been exposed to a sterile non-lubricated vaginal speculum. The microbiological examinations of all cultures were performed by the same laboratory staff throughout the entire study and the clinical findings were blinded.

All microbiological methods have been described in details previously [4]; however, methods behind the variables used in the analyses of this paper are described below.

The clinical diagnosis of BV, as defined by Amsel et al [11], required that three out of the following four criteria be present: (1) fluid from the top of the vagina with a pH of > 4.5, (2) homogeneous adherent discharge, (3) clue cells on the saline wet mount, and (4) fishy odor after addition of 10% potassium hydroxide to the discharge (amine test). A saline (0.9% sodium chloride solution) wet mount was made for instant direct microscopy. To perform the amine test a vaginal wash with 2 ml sodium chloride solution (0.9%) was taken and 10% potassium hydroxide was added to the specimen. The pH value of vaginal discharge was determined by colorimetric pH-paper (pH-indicator strips, manufactured by E. Merck, Germany) with ten comparison colors for pH values between 4.0 and 7.0.

*Lactobacillus* spp were identified as gram-positive rods with a typical colonial appearance. *Lactobacillus* spp were not identified at genus level. Detection was reported as 1+, 2+, and 3+, indicating the streak zones.

Material from charcoal swabs was plated onto chocolate agar plates with 7% previously reduced horse blood, vitamin K (1.0 mg/L), and cysteine (550 mg/L) for cultivation and isolation of anaerobic bacteria. The plates were incubated in an anaerobic chamber at 35°C for 4 days. The term *Bacteroides* spp includes those isolates now known as *Prevotella* spp and *Porphyromonas* spp and nonspecific anaerobic bacteria include isolates of *Bifidobacterium* spp, *Peptostreptococcus* spp, *Propionibacterium* spp, *Clostridium* spp, and *Veillonella* *parvula* among others.

### Statistical analyses

Pearson's χ^2^-test was used for comparisons of the proportional distributions. P-values below .05 were regarded as statistically significant. The software package SPSS was used for data analyses. For the purpose of estimating an association between *Lactobacillus* spp growth and elimination and acquisition of BV, a new variable was defined and dichotomized as heavy *Lactobacillus* spp growth (streak zones 2+ and 3+) or not. Likewise smoking in pregnancy was dichotomized as smoking more than 10 cigarettes at enrollment.

## RESULTS

The 229 women in this study were not different in respect to prevalence of BV, age at estimated date of delivery, pre-pregnancy weight, gestational weight gain, height, body mass index (BMI), *Lactobacillus* spp, sexual activity, and smoking at enrollment from the entire cohort (*n* = 2927) and non-participants (*n* = 39). However, the 229 women included were examined 1 week earlier at enrollment (mean gestational age: 16*w* + 0*d* versus 17*w* + 2*d*/17*w* + 1*d*, *P* < .05). Four participants delivered preterm.

BV seemed to decrease from 17% at enrollment to 14% in mid-third trimester (Wilcoxon, *P* = .13). This reduction in prevalence was due to 39% (14/38) of the cases who were BV positive at enrollment eliminated BV spontaneously until mid-third trimester (Table 1), while only 4% (7/191) of those women who initially were tested negative for BV acquired BV from enrollment to mid-third trimester (Pearson's χ^2^-tes *t* = 97, *P* < .001). No statistically significant differences in the prevalence of the clinical criteria used for diagnosing BV at enrollment and in mid-third trimester (32*w* + 3*d*) were found: characteristic vaginal discharge (14 versus 14%), pH above 4.5 (32 versus 27%), Amine test positive (21 versus 18%), and clue cells (25 versus 30%), (*N* = 229).

Risk factors at enrollment for the acquisition of BV were explored in spite of limited number of cases acquiring BV during pregnancy (*n* = 7). Heavy smokers were at increased risk for the acquisition of BV during pregnancy, as were women receiving public benefits (Table 2). A previous use of combined oral contraceptives was preventive for the acquisition of BV. Women with a vaginal pH above 4.5 or having vaginal anaerobe bacteria (specific or nonspecific) were also at increased risk for the acquisition of BV during pregnancy (Table 3).

Factors at enrollment associated with an increased chance of spontaneously eliminating of BV (*n* = 24) were also explored. Only women with a heavy growth of *Lactobacillus* at enrollment (streak 2+ or over) tended to have a greater chance of eliminating BV spontaneously (Table 3).

Elimination or acquisition was not significantly associated with the following risk factors: alcohol consumption, change of partner during pregnancy, not-cohabitant/single, coital frequency, a previous pelvic inflammatory disease, or any of the other risk factors defined. Further, neither elimination nor acquisition was significantly associated with the following other microorganisms: *M. hominis, U. urealyticum, G. vaginalis*, or other microorganisms evaluated.

## DISCUSSION

This is the first longitudinal study of BV in pregnancy using Amsel's clinical criteria for BV. Two hundred twenty-nine pregnant women, a random sample of a population-based study on 2927 women, were followed. BV prevalence decreased from 17% early in the second trimester to 14% in mid-third trimester due to a tenfold higher rate of elimination among BV-positive subjects (39%) in comparison with the acquisition of BV among BV-negative subjects (4%) during the same observational period. However, no statistically significant differences in the prevalence of the clinical criteria used for diagnosing BV at enrollment and in mid-third trimester (32*w* + 3*d*) were found. The risk of acquiring bacterial vaginosis during pregnancy is low, however, higher among heavy smokers and public benefit recipients. Further, women with a pH above 4.5 or anaerobe bacteria (specific or unspecific) at enrollment had an increased risk of acquiring bacterial vaginosis. Women with heavy growth of *Lactobacillus* at enrollment tended to have a greater chance of spontaneously eliminating BV in pregnancy.

The prevalences of bacterial vaginosis in this pregnancy subset of women is comparable to other Scandinavian populations. In Finland a prevalence of 21% was found in 1992 among healthy nulliparous examined week 8–17 [12] whereas in 2001 it was reported to be 10% in a low-risk Finnish group examined week 10–17 [13]. The reduction in prevalence in our study is in consistent with the findings by Riduan et al [7], Hay et al [7], and Platz-Christensen et al [6]. In the multicenter study by Hillier et al [2] BV prevalence increased, which could potentially be due to a higher proportion of African-American (38%), who have a higher prevalence of BV in general (23%) as well as different behavior such as douching [14].

In this study a number of demographic, behavioral, and reproductive risk factors were examined. The current study found smoking to be a significant risk factor for acquiring BV in pregnancy. Smoking is a known risk factor for having BV in pregnancy [15, 16]. Hellberg et al [17] and Smart et al [18] have both found a dose response relationship between smoking and BV in non-pregnant women, possibly indicating a causal pathway. The effect of smoking on acquisition of BV could be due to a reduction in the placenta's ability to produce estrogens [19, 20], a factor which again could result in decreased growth of *Lactobacillus* spp. However, smoking is also known to be a social class indicator, and other indicators of social class such as public benefit recipients were also found to be associated with acquisition of BV.

Sexual activity or change of partner seemed not to affect either acquisition or elimination of BV. Sexual activity has been suggested to be associated with the acquisition of BV also in pregnancy [5]. The difference in these results may potentially be explained by the limited sample size of the present study. The previous use of oral contraceptives was found to be preventive of the acquisition of BV in pregnancy. This could be interpreted as a selection bias towards healthy young women with spontaneously conceived pregnancies.

The study also allowed for evaluation of microbiological factors of vaginal flora and the risk of acquiring or eliminating BV in pregnancy. We found that heavier growth of *Lactobacillus* spp tended to increase chances for spontaneous elimination of BV. The negative association between *Lactobacillus* spp and BV is well established from previous studies [8, 21] as well as in the total cohort [4] of which the current study is a subset. Hawes et al. followed 182 women in a STD clinic during a two-year period and demonstrated that women without *Lactobacillus* spp and women with non-H_2_O_2_ producing *Lactobacillus* spp have an increased risk of acquiring BV. Due to the increased growth of H_2_O_2_ producing *Lactobacillus* vaginal pH will decrease and result in deteriorated conditions for the BV-associated microflora. Hillier et al. found the 61% of pregnant women without BV to have H_2_O_2_ producing *Lactobacillus* spp compared to only 5% in the women with BV, whereas the H_2_O_2_ negative lactobacilli were equally frequent [21]. The early observation of increased growth of *Lactobacillus* spp among women who spontaneously eliminate their BV could indicate a slow transformation from BV positivity towards negativity involving an intermediate stage as demonstrated in longitudinal studies [5, 8] using the Gram stain methods. An intermediate stage towards BV could also explain why we find women with a vaginal pH above 4.5 and women with anaerobe bacteria to be at increased risk for acquiring BV during pregnancy. These women do not yet fulfill 3 out of 4 criteria for BV, but have the most common BV criteria pH > 4.5, present in the current study in 1/3 women, or the associated microflora such as anaerobe bacteria.

The present study is a longitudinal investigation from a population-based study. The 229 women in the study were comparable to the entire cohort and to the non-participants exept for gestational age at enrollment. The fact that participants selected for two examinations were enrolled one week before the other groups may decrease generalizability of data to the entire cohort, as BV was affected by gestational age. Amsels clinical criteria for diagnosing BV were used, and most examinations (98%) were performed by one doctor. The clinical findings were blinded for all clinical staff during the completion of the study. Additional methods of diagnosis as Nugent or Spiegel would have provided interesting comparisons. Information on sexual behavior and smoking habits was obtained from questionnaires fulfilled by the women at home prior to the prenatal care visits; however, life-style data (interview or questionnaire) always pose a substantial risk for misclassification [22]. Yet, sample size is without doubt the most important problem when interpreting validity of the current results.

The findings suggest that acquisition of BV during pregnancy is rare and is associated with smoking, low social class (defined as public benefit recipients), the presence of anaerobe bacteria or a vaginal pH > 4.5. Heavy growth of *Lactobacillus* spp in early pregnancy tended to be an indicator of women on the way to eliminate their BV. However, the sample size for studying acquisition and elimination of BV is very small and the observations should be interpreted with caution.

## CONDENSATION

Prevalence of bacterial vaginosis (clinical criteria) in pregnancy decreases as the pregnancy progresses. The risk of acquiring bacterial vaginosis during pregnancy is low, however, higher among heavy smokers, public benefit recipients, and among women with a pH above 4.5 or with anaerobe bacteria at enrollment. Elimination of BV in pregnancy only tended to be associated with heavy growth of Lactobacillus.

## Figures and Tables

**Table 1:** Dynamics of bacterial vaginosis (BV) diagnosed by Amsel's criteria from enrollment until delivery (*N* = 229).

BV		Midtrimester			Odds ratio
		32*w* + 3*d*			95% CI

		+	−	Total
Enrollment	+	24	14	38	46
16*w* + 0*d*	−	7	184	191	[16–124]
Total		31	198	229

**Table 2:** Behavioral risk factors for the acquisition or elimination of bacterial vaginosis (Amsel's criteria) in pregnancy. (*N* = 229.)

Exposure at enrollment		Acquisition of BV			Elimination of BV		

	+	−	^†^OR [95% ^‡^CI]	+	−	^†^OR [95% ^‡^CI]	
Smoking > 10/*d*	+	4	37	**5.3 [1.1–25]**	3	7	0.7 [0.14–3.1]
	−	3	147		11	17	
Sex > 2/w^#^	+	3	92	0.7 [0.2–3.4]	8	10	1.0 [0.3–4]
	−	4	90		6	13	
No basic education^□^	+	1	4	7.5 [0.7–77]	0	1	—
	−	6	180		14	23	
Previous use of COC^$^	+	2	127	**0.2 [0.03–0.96]**	8	18	0.4 [0.1–1.8]
	−	5	57		6	6	
Physically demanding	+	3	30	3.9 [0.8–18]	0	4	—
job*	−	4	154		14	20	
Public benefit	+	4	40	**4.8 [1.0–22]**	8	11	1.6 [0.4–5.9]
Recipients	−	3	144		6	13	
Alcohol > 4/*w* ^#^	+	1	3	10 [0.9–111]	0	0	—
	−	6	180		14	24	

^†^OR: odds ratio, ^‡^CI: confidence interval. ^#^Sexual activity were measured as coitus frequency for the four weeks prior to fulfillment of the questionnaire.

^□^No higher education was defined as not having pursued formal education past the ninth grade . ^$^Any previous use of combined oral contraceptives.

*A physically demanding job was defined as a job belonging to the highest of 4 categories on the basis of physical workload. ^#^4 or more drinks per week at enrollment.

**Table 3:** Microbiological risk factors for the acquisition or elimination of bacterial vaginosis (Amsel's criteria) in pregnancy. (*N* = 229).

Exposure at enrollment		Acquisition of BV			Elimination of BV		

		+	−	OR [95% CI]	+	−	OR [95% CI]
Heavy							
*lactobacillus*	+	5	142	0.7 [0.14–3.9]	8	7	3.2 [0.8–13]
growth^#^									
	−	2	42		6	17	
Anaerobe bacteria	+	2	4	**18 [2.7–122]**	8	17	0.5 [0.1–2.1]
Nonspecific	−	5	180		6	7	
Anaerobe bacteria	+	2	7	**10 [1.7–62]**	8	17	0.5 [0.1–2.1]
Specific	−	5	177		6	7	
Vaginal pH > 4.5	+	14	24	**6.3 [1.4–29]**	14	24	—
		0	0		0	0	

Growth of *Lactobacillus* spp was reported as 1+, 2+, and 3+ indicating the streak zones. ^#^defined as 2+ and above. The term specific anaerobe includes *Bacteroides spp, Prevotella spp* and *Porphyromonas spp* and nonspecific anaerobic bacteria include isolates of *Bifidobacterium spp, Peptostreptococcus spp, Propionibacterium spp, Clostridium spp*, and *Veillonella parvula* among others.
